# Reassessing the risks of Cervical Transforaminal Epidural Steroid Injections (CTFESI)

**DOI:** 10.1016/j.inpm.2025.100625

**Published:** 2025-08-15

**Authors:** Clark C. Smith

**Affiliations:** Columbia University, Vagelos College of Physicians and Surgeons, USA

The landscape of interventional pain medicine is continuously evolving, driven by a commitment to both efficacy and patient safety. Within this dynamic field, cervical transforaminal epidural steroid injections (CTFESI) have traditionally held a uniquely cautious position. Recent empirical data is now reshaping this view, prompting a critical reassessment of procedural risks, particularly concerning the management of antiplatelet and anticoagulant medications.

The influential 2017 and 2020 large observational studies by Endres et al. [1,2], while primarily focused on lumbar procedures, demonstrated that the risks of withholding vital antithrombotic medications (such as stroke or myocardial infarction) must be carefully weighed against the risks of continuing them during procedures. This call for a nuanced, individualized approach has been actively embraced by leading societies like the International Pain and Spine Intervention Society (IPSIS) and the American Society of Regional Anesthesia and Pain Medicine (ASRA), who have since updated their risk stratification guidelines for various interventional spine procedures. This evolving understanding is now further bolstered by emerging real-world data specifically related to CTFESI.

Historically, the anatomical confines of the cervical intervertebral foramen and the proximity to critical structures like the spinal cord and vertebral artery have led to heightened concerns about potential catastrophic complications from CTFESI, especially bleeding events such as spinal cord compression from an epidural hematoma. Despite the scarcity of robust evidence quantifying this specific risk in anticoagulated patients, this perceived elevated risk frequently led to the discontinuation of antiplatelet or anticoagulant therapy, potentially exposing patients to significant systemic thrombotic events.

It is within this crucial context that recent studies offer important insights, challenging assumptions about the bleeding risks and overall safety of CTFESI. The retrospective review by Levi et al. [3] provides compelling real-world data on the safety profile of these procedures in anticoagulated patients. Their observational study included over 1000 injections performed on patients who continued some form of anticoagulant, aspirin (ASA), or NSAID medication. Crucially, the authors reported zero cases of symptomatic epidural hematomas or other bleeding complications in the immediate post-procedural period or up to one week following the injection.

The low overall complication rate for CTFESI is further reinforced by the large national database study from Stephens et al. [4]. Analyzing data from 32,913 patients, they found the risk of major complications to be exceptionally low: symptomatic epidural hematomas were ≤0.03 %, cerebral infarction codes were 0.15 %, seizures 0.12 %, and paralysis 0.5 %. There were no mortality-related codes within a day after CTFESI. While this study does not specifically analyze anticoagulated patients, its findings support the notion that the procedure itself may carry a lower inherent risk than previously assumed.

Recent evidence also provides robust data supporting the safety of CTFESIs when non-particulate steroids are used. A large study by Beckworth et al. [5] reviewed 6241 cervical TFESIs performed over a 17-year period and reported no catastrophic complications (such as spinal cord injury, stroke, or death). A similar large retrospective cohort study by Wozniak et al. [6] of 1018 CTFESI procedures using non-particulate steroids also found no major complications. This substantial and consistent real-world data directly supports the safety of CTFESI under modern protocols.

The findings from these recent studies collectively suggest that the perceived bleeding risk of CTFESI, particularly in the context of carefully managed anticoagulation and adherence to modern safety protocols, may be lower than previously feared. This reassessment has naturally prompted further discussions regarding the overall safety of the procedure. In Interventional Pain Medicine, Narouze [7] compellingly advocates for “reclaiming confidence in cervical transforaminal epidurals.” Narouze emphasizes that the 2014 FDA warning was primarily linked to particulate steroids and asserts that CTFESI can be safely performed with strict adherence to safety measures—including the use of non-particulate steroids, real-time fluoroscopy with contrast injection, and pre-procedure MRI review for vertebral artery anatomy. Furthermore, Narouze challenges the perception that cervical interlaminar epidural steroid injections (CILESI) are inherently safer, citing malpractice claims and case reports that demonstrate significant complications associated with CILESI.

The findings of these recent studies provide a foundation for a comprehensive reassessment of risks associated with CTFESI. They also foster an increasingly evidence-based, patient-centric approach to anticoagulation management in interventional spine procedures. Physicians performing spine procedures must diligently balance the bleeding risk of continuing antiplatelet and anticoagulant medications against the very real and potentially life-threatening cardiovascular and cerebrovascular events associated with withholding those medications. Further large-scale, prospective studies are warranted to definitively confirm these important results and continue to refine our understanding.

## Declaration of competing interest

The authors declare that they have no known competing financial interests or personal relationships that could have appeared to influence the work reported in this paper.
